# Evaluation of Effectiveness and Tolerability of Saroglitazar in Metabolic Disease Patients of India: A Retrospective, Observational, Electronic Medical Record-Based Real-World Evidence Study

**DOI:** 10.7759/cureus.89028

**Published:** 2025-07-30

**Authors:** Sambit Das, Sunil Gupta, Tejal Lathia, Jayshree Swain, Sachin Mittal, Sharad Kumar, Mahesh DM, Neeraj Garg, Ravi Teja, Anne Beatrice, Basavaraj G Sooragonda, Syed Mohd. Razi, Ashok Jaiswal, Kunal S Jhaveri, Snehal Shah, Garima Verma

**Affiliations:** 1 Endocrinology, Kalinga Institute of Medical Sciences, Bhubaneswar, IND; 2 Diabetology, Sunil's Diabetes Care n' Research Centre, Nagpur, IND; 3 Endocrinology, Apollo and Cloud Nine Hospitals, Navi Mumbai, IND; 4 Endocrinology, Diabetes and Metabolism, Institute of Medical Sciences and Sum Hospital, Bhubaneswar, IND; 5 Endocrinology, Care Plus Clinic, Chandigarh, IND; 6 Endocrinology, Hormonal Center, Lucknow, IND; 7 Endocrinology, Aster Corporate Hospital, Bengaluru, IND; 8 Endocrinology, Endoderma World Clinic, Mohali, IND; 9 Endocrinology, Advanced Centre for Endocrinology and Diabetes (ACE), Visakhapatnam, IND; 10 Endocrinology, Nizam’s Institute of Medical Sciences, Hyderabad, IND; 11 Endocrinology, Diabetes and Metabolism, Narayana Hrudayalaya Hospitals, Bengaluru, IND; 12 Endocrinology, Sri Sai Super Speciality Hospital, Moradabad, IND; 13 Medical Affairs, Zydus Lifesciences, Mumbai, IND; 14 Insights, HealthPlix Technologies, Bengaluru, IND; 15 Real-World Evidence, HealthPlix Technologies, Bengaluru, IND

**Keywords:** glycosylated hemoglobin, metabolic diseases, real-world, renal impairment, saroglitazar, triglycerides

## Abstract

Background

Metabolic disorders, including diabetes mellitus (DM), diabetic dyslipidemia (DD), and metabolic-dysfunction-associated steatotic liver disease (MASLD), are significant health challenges in India. This study aims to evaluate the real-world effectiveness and tolerability of saroglitazar (4mg) in Indian adults with type 2 diabetes mellitus (T2DM), DD, and MASLD, focusing on changes in glycemic, lipid, and hepatic biomarkers.

Methods

This retrospective study included adult patients with metabolic diseases (≥ 18 years) who were prescribed saroglitazar (4 mg) at baseline and continued therapy at least till the next follow-up visit after 90 days. The patients with at least one follow-up visit after 90 days from baseline with values for glycemic parameters, lipid parameters, aspartate transaminase (AST), and alanine transaminase (ALT) available at both visits were included. Changes in glycemic parameters, lipid profile, and liver enzymes were assessed from baseline to the follow-up visit. Disease conditions, concomitant medications at baseline, and adverse events at the follow-up visit were also evaluated.

Results

A total of 553 patients were included in this study. The most common conditions at baseline were DM, dyslipidemia, and hypertension. Saroglitazar significantly improved glycemic control, reducing glycosylated hemoglobin (HbA1c) by -0.71% from baseline to the follow-up visit. Fasting blood glucose (FBG) and postprandial blood glucose (PPBG) in the overall patient population decreased by -21.24 mg/dL (n =410) and -24.28 mg/dL (n = 178), respectively. In patients with baseline FBG >100 mg/dL (n = 358), the FBG reduction was −26.46 mg/dL, while in patients with PPBG >140 mg/dL (n = 151), the PPBG reduction was −31.73 mg/dL. There was a substantial improvement in the lipid profile, including a significant reduction in serum triglycerides (TG) (−55.41 mg/dL) and LDL (−6.95 mg/dL). Hepatic parameters improved, with AST and ALT decreasing by −2.62 IU/L and −7.95 IU/L, respectively. No significant adverse events and renal impairment were observed.

Conclusion

Saroglitazar demonstrated significant improvements in glycemic control, lipid profile, and liver enzymes with a favorable safety profile in Indian patients with metabolic diseases.

## Introduction

Metabolic disorders, encompassing diabetes mellitus (DM), diabetic dyslipidemia (DD), and metabolic-dysfunction-associated steatotic liver disease (MASLD, formerly non-alcoholic fatty liver disease [NAFLD]), are significant public health challenges worldwide and are increasingly prevalent in India [1]. These interrelated conditions are driven by intricate mechanisms such as insulin resistance, chronic low-grade inflammation, and lipid abnormalities, which significantly elevate the risk of cardiovascular complications and other morbidities [2]. Globally, the prevalence of metabolic syndrome varies widely depending on diagnostic criteria, geography, ethnicity, and demographic factors [3,4]. In India, metabolic syndrome affects approximately 30% of the population, with higher rates observed among women, urban residents, and individuals over 60 years of age [5].

This rising burden of metabolic disorders in India is further exacerbated by lifestyle changes, including obesity, physical inactivity, and unhealthy dietary patterns linked to socio-economic shifts and the phenomenon of "nutrition transition" [6,7]. Despite efforts focusing on lifestyle modifications and pharmacological interventions, achieving comprehensive metabolic control and preventing long-term complications of metabolic disorders remain formidable challenges [8]. This emphasizes the critical need for novel, effective, and well-tolerated therapeutic options tailored to address the complex interplay of metabolic abnormalities in the Indian population.

The metabolic diseases are generally treated with anti-obesity, anti-diabetic, lipid-lowering, and anti-hypertensive drugs with central nervous system and peripheral actions, leading to significant polypharmacy concerns [8]. This highlights the necessity for novel therapeutic strategies targeting multiple metabolic disease components to provide a comprehensive treatment approach [9]. Research has demonstrated that dual peroxisome proliferator-activated receptor (PPARα/γ) agonists can address this gap by modulating both lipids and glucose profiles through complementary mechanisms [10,11].

In this context, saroglitazar, a dual PPAR α/γ agonist, has emerged as a promising drug that is approved for managing indications like diabetic dyslipidemia (DD), MASLD, and type 2 diabetes mellitus (T2DM). Its dual action enhances insulin sensitivity, regulates adiponectin and leptin levels, induces fatty acid β-oxidation, and reduces lipotoxicity-mediated oxidative stress [12]. Various clinical and preclinical studies in India demonstrated the effectiveness of saroglitazar in improving the lipid profile and achieving optimal glycemic control for the treatment of dyslipidemia and diabetes [13,14]. Notably, a recent clinical study also highlighted its potential in improving atherogenic dyslipidemia and lipoprotein profile irrespective of comorbidities in patients with MASLD [15]. Furthermore, a clinical case series enumerated the significant benefits of saroglitazar in considerably improving glycemic control, lipid profile, liver enzymes, liver fat, and fibrosis in MASLD patients [16]. Interestingly, saroglitazar has also shown promising effects in treating MASLD and metabolic dysfunction-associated steatohepatitis (MASH) not only in diabetic patients but also in non-diabetic individuals and even in patients with compensated cirrhosis [17]. Its ability to target multiple metabolic pathways with a favorable safety profile makes saroglitazar a promising option for managing complex metabolic conditions [18].

Owing to the limited real-world data on the clinical utility of saroglitazar, this retrospective study aims to evaluate its effectiveness and tolerability in managing metabolic disorders, including T2DM, DD, and MASLD, in Indian patients. Accordingly, this study evaluates changes in key clinical parameters, including HbA1c, fasting and postprandial blood glucose (FBG and PPBG), lipid profile, liver enzymes, and renal function among Indian patients receiving saroglitazar. This is the first electronic medical records (EMR)-based real-world study on saroglitazar (Lipaglyn® 4mg) to cumulatively evaluate its effectiveness along with safety and tolerability in Indian patients (LEAD INDIA EMR). The findings aim to offer valuable insights into the therapeutic potential of saroglitazar in addressing the multifaceted challenges of metabolic diseases.

## Materials and methods

Study design

This retrospective observational study analyzed the anonymized electronic medical records (EMR) to evaluate the effectiveness of saroglitazar in Indian patients with metabolic diseases.

This study included male and female patients ≥18 years with at least one metabolic disorder (MASLD/T2DM/dyslipidemia/obesity) mentioned in the EMR who were prescribed saroglitazar. The above patients with values of glycosylated hemoglobin (HbA1c), aspartate transaminase (AST), alanine transaminase (ALT), low-density lipoprotein (LDL), and serum triglycerides (TGs) available at baseline (visit 1) and at a follow-up visit (visit 2) were included for analysis. The baseline visit was the visit in which saroglitazar was prescribed. Any visit 90 days after the initiation of saroglitazar was considered as the follow-up visit (visit 2). The patients who did not meet the above criteria were excluded from the study.

The primary objectives were to evaluate the changes in the levels of HbA1c, LDL, TG, AST, and ALT from baseline to visit 2. It also included the analysis of the concomitant medications and the conditions at baseline. The secondary objectives were the assessment of change in the values of high-density lipoproteins (HDL), total cholesterol (TC), non-HDL, FBG, and PPBG from baseline to visit 2. Similarly, the sub-analysis of the liver enzymes was done to understand saroglitazar’s effect in patients with diseased liver conditions. Additionally, renal safety was also determined by evaluating the changes in the serum creatinine and estimated glomerular filtration rate (eGFR).

Data collection and variables

Anonymized and aggregated data of the patients from January 2017 to October 2024 were retrieved from the HealthPlix EMR database for analysis [34]. The study was approved by the Central Independent Ethics Committee (CIEC), Pune (IEC No.: CIEC231224, dated 17th December 2024). The data required for the study were retrieved from the HealthPlix database on 20th December 2024.

HealthPlix is a practice management software used by over 14,000 doctors across India to write prescriptions. De-identified patient data was retrieved in an Excel format (Microsoft, Redmond, USA) and cleansed as per HealthPlix's standard operating procedure (SOP). Patient confidentiality was maintained throughout the study. Baseline data (visit 1) was analyzed to understand demographic characteristics, including baseline age, gender, weight, and body mass index (BMI). Data on baseline conditions and concomitant medications were retrieved and analyzed. The baseline and follow-up visit (visit 2) data were evaluated to understand the change in glycemic parameters, lipid profile, and liver enzyme levels. Since this study involved analysis of retrospective anonymized, de-identified data, there was a waiver of patient consent. The comorbid conditions mentioned in the study were defined by the doctors and entered into the EMR fields.

Statistical analysis

StataCorp. (2017). Stata Statistical Software: Release 15.1 Limited Edition, TX: StataCorp LLC. was used for statistical analysis for this study. Categorical variables, such as gender, were summarized as counts (n) and percentages. Continuous variables, including age, weight, TC, HDL, non-HDL cholesterol, LDL, TG, HbA1c, FBG, PPBG, AST, and ALT, were described using descriptive statistics. These include the number of patients (n), mean, standard deviation (SD), quartiles, minimum and maximum values, and 95% confidence intervals (CIs). Changes in laboratory parameters over time were analyzed for statistical significance using either the paired Student's t-test or the Wilcoxon signed-rank test, depending on the distribution of the variable. The missing values were not imputed.

## Results

Patient disposition

**Figure 1:**
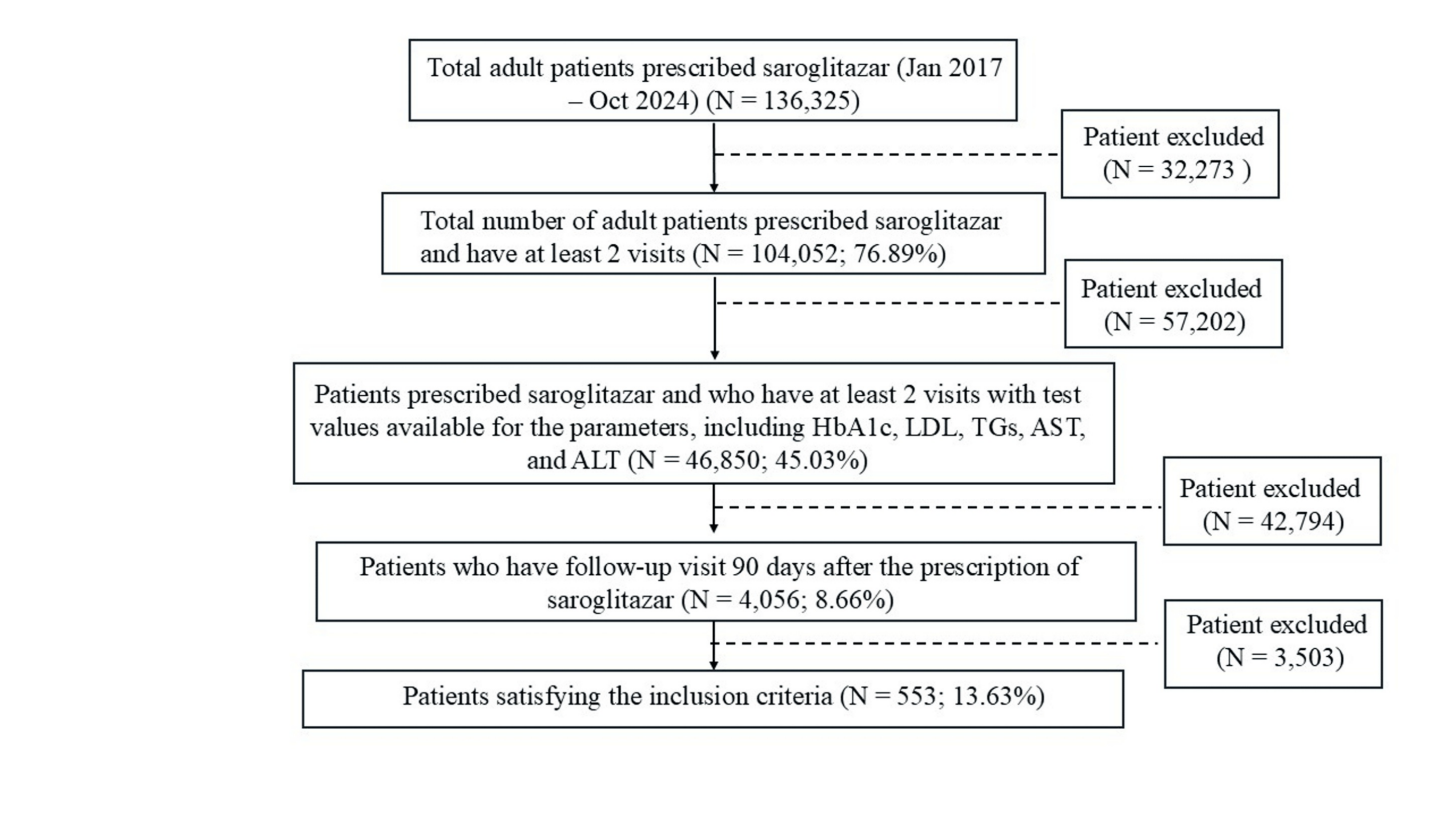
CONSORT diagram N: Number of patients, HbA1c: Glycosylated hemoglobin, LDL: Low-density lipoprotein, AST: Aspartate transaminase, ALT: Alanine transaminase

Data of 1,363,25 adult patients prescribed saroglitazar were available in the EMR (Jan 2017 to Oct 2024). Of these 1,040,52 (76.89%) of patients had at least two visits recorded in the EMR. Among these patients, the laboratory values for HbA1c, AST, ALT, LDL, and TGs were available for 468,50 (45.03%) patients. Within this group, 4,056 (8.66%) of patients had follow-up visits 90 days after the prescription of saroglitazar. Furthermore, 553 (13.63%) patients met the eligibility criteria. The patient disposition is illustrated in the CONSORT diagram (Figure 1).

Baseline characteristics

The data of 553 patients prescribed saroglitazar and who had HbA1c, LDL, TG, AST, and ALT values at baseline and at the follow-up visit were retrieved and analyzed. The mean age of the patients was 50.72±11.26 years, with a majority belonging to the 18-49 years age group (N=262, 47.38%). Males constituted 68.90% (N=381) of the total 553 patients. Data on body weight were available for 441 patients, and their mean weight was 78.33 ± 12.86 kg. For the 361 patients with recorded BMI, the mean BMI was 29.08 ± 4.31 kg/m². Most of the patients (N=309, 85.61%) were obese (BMI ≥ 25.00 kg/m²). The median duration of the treatment (N= 553) in the study was 439.41 ± 371.51 days (Table 1).

**Table 1:** Baseline characteristics The data has been represented as N (%) and Mean±SD. N: Number of patients, BMI: Body Mass Index, SD: Standard Deviation

Baseline characteristics			
Demographics		N (%)	Mean ± SD
Age (years)	Overall	553 (100)	50.72 ± 11.26
	18 – 49 years	262 (47.38)	40.90 ± 5.74
	50 – 59 years	165 (29.84)	54.67 ± 2.76
	≥60 years	126 (22.78)	65.94 ± 5.06
Gender	Overall	553 (100)	NA
	Male	381 (68.90)	NA
	Female	172 (31.10)	NA
Weight (kg)	Overall	441 (100)	78.33 ±12.86
BMI (kg/m^2^)	Overall	361 (100)	29.08 ± 4.31
	Underweight (<18.50)	2 (0.55)	17.16 ± 1.59
	Normal (18.50 to 22.99)	12 (3.32)	21.29 ± 1.13
	Overweight (23.00 to 24.99)	38 (10.53)	24.01 ± 0.62
	Obese (≥25.00)	309 (85.61)	30.09 ± 3.76
Median duration of treatment (Days)			
N (%)	Median ± SD		
553	439.41 ± 371.51		

DM was the most prevalent condition, reported for 90.24% (N=499) of the study population. Dyslipidemia/hypertriglyceridemia (N=261, 47.20%) and hypertension (N=256, 46.29%) were the other prominent conditions present in the patients prescribed saroglitazar. Patients also had double comorbidities and triple comorbidities, of which diabetes and dyslipidemia (N=248, 44.58%) and dyslipidemia, hypertension, and DM (N=118, 21.34%) were profoundly seen (Table 2).

**Table 2:** Conditions at baseline The data has been represented as N (%). DM: Diabetes Mellitus, NAFLD: Non-alcoholic Fatty Liver Disease, MASLD: Metabolic Associated Steatohepatitis Leading to Liver Disease

Conditions	N	%
Diabetes mellitus/impaired fasting sugar/Hyperglycemia/DM	499	90.24%
Dyslipidemia/hypertriglyceridemia	261	47.20%
Hypertension	256	46.29%
Non-alcoholic fatty liver disease/fatty liver/MASLD/transaminitis	143	25.86%
Diabetes+Dyslipidemia	248	44.85%
Diabetes+ Hypertension	234	42.31%
Hypertension + Dyslipidemia	122	22.06%
Dyslipidemia + Hypertension + DM	118	21.34%

Concomitant medications

The data was analyzed to evaluate the co-prescribed medications. The antidiabetic, antihyperlipidemic, and antihypertensive drugs were the most recommended. The other concomitant medications included vitamin D supplements, proton pump inhibitors, anti-gout medications, non-selective cyclooxygenase (COX) inhibitors, antidepressants, drugs for benign prostate hyperplasia, medications for neuropathic pain and kidney ailments, and antifungals. Among the antidiabetic agents, biguanides were mostly recommended. A combination of dipeptidyl peptidase 4 (DPP4) inhibitors and metformin was frequently recommended as a dual antidiabetic medication. Among the antihyperlipidemic agents and antihypertensives, statins and angiotensin II receptor blockers (ARBs) were the most frequently prescribed (Table 3).

**Table 3:** Concomitant medications at baseline *The patients can be on more than one medication. The data has been represented as N(%). N: Number of patients, SGLT2: Sodium-glucose cotransporter-2, DPP4: Dipeptidyl peptidase-4, ARB: Angiotensin receptor blockers, CCB: Calcium channel inhibitors, ACE: Angiotensin-converting enzyme

Drug class	Therapy type	Medications^*^	N	%
Antidiabetic agents	Single agent	Biguanides	57	5.30
		Insulin Glargine	44	4.09
		SGLT2 Inhibitors	41	3.81
		DPP4 Inhibitors	82	7.62
		Sulfonylureas	40	3.72
		Insulin Degludec	20	1.86
	Dual agent	DPP4 inhibitors + Metformin	161	14.96
		Sulfonylureas + Metformin	182	16.92
		SGLT2 + Metformin	29	2.70
		SGLT2 + DPP4 inhibitors	26	2.42
		Insulin Aspart + Insulin Degludec	21	1.95
	Triple agent	Glimepiride + Metformin + Voglibose	39	3.62
		Dapagliflozin + Metformin + Sitagliptin	25	2.32
	Combination insulin	Human Insulin + Nph Human Insulin	21	1.95
Antihyperlipidemic agents	Single agent	Statins	215	63.42
	Dual agent	Fenofibrate + Statin	40	11.80
		Aspirin + Statins	62	18.29
		Clopidogrel + Statin	11	3.24
		Ezetimibe + Statin	6	1.77
		Statin + ARB	5	1.47
Antihypertensives	Single agent	ARB	73	31.6
		Beta-blockers	42	18.18
		CCB	27	11.69
		ACE inhibitors	6	2.60
	Dual agent	CCB + ARB	38	16.45
		Beta-blockers + ARB	17	7.36
		Chlorthalidone + ARB	12	5.19
		Hydrochlorothiazide + ARB	6	2.60
		CCB + Atenolol	5	2.16
	Triple agent	CCB + Hydrochlorothiazide + Angiotensin II receptor antagonists	5	2.16

Effectiveness of saroglitazar

Change in Glycemic Parameters

Any visit 90 days after the initiation of saroglitazar was considered the follow-up visit. Analysis of the HbA1c levels in the overall population (N=553) revealed a statistically significant reduction of -0.71% from a baseline value of 8.20±1.76% to 7.49±1.50% at the follow-up visit (p<0.001). Among patients with HbA1c ≥ 6.5% at baseline (N=472), a statistically significant mean reduction of -0.87% (p<0.001) was observed. Similarly, those with a baseline HbA1c ≥7% (N=405) and ≥7.5% (N=349) also showed statistically significant reductions of -1.05% and -1.21%, respectively (p<0.001).

FBG levels significantly decreased from baseline to follow-up, with a greater reduction observed in patients with higher initial glucose levels. Similarly, PPBG levels showed a notable decline, with a more substantial reduction among those with elevated baseline values. These findings underscore the effectiveness of saroglitazar in improving glycemic control, particularly in patients with higher baseline glucose levels, supporting its role in managing diabetes (Table 4).

**Table 4:** Change in glycemic parameters p-value calculated using Signed Rank-Sum at 5% level of significance.***p-value significant at 0.001 level of significance. The data has been represented as N, Mean±SD. HbA1c: Glycated Hemoglobin, DM: Diabetes Mellitus, FBG: Fasting Blood Glucose, PPBG: Post Prandial Blood Glucose, SD: Standard Deviation

Parameter	N	Baseline (Mean ± SD)	Follow-up (Mean ± SD)	Mean change	p-value^***^	z value
HbA1c (%)						
Overall	553	8.20 ± 1.76	7.49 ± 1.50	-0.71	<0.001	-9.854
HbA1c<5.7% at Baseline	17	5.34 ± 0.34	5.63 ± 0.69	0.29	0.0872	1.710
HbA1c (5.7 to 6.4%) at Baseline	64	6.10 ± 0.22	6.24 ± 0.67	0.14	0.2724	1.098
HbA1c ≥6.5% at Baseline	472	8.60 ± 1.62	7.73 ± 1.48	-0.87	<0.001	-10.524
HbA1c ≥7% at Baseline	405	8.90 ± 1.53	7.85 ± 1.50	-1.05	<0.001	-11.204
HbA1c ≥7.5% at Baseline	349	9.18 ± 1.47	7.97 ± 1.54	-1.21	<0.001	-11.242
FBG (mg/dL)						
Overall	410	152.13 ± 52.13	130.89 ± 46.35	-21.24	<0.001	-7.929
>100 mg/dL at Baseline	358	161.24 ± 49.48	134.78 ± 46.62	-26.46	<0.001	-8.848
PPBG (mg/dL)						
Overall	178	210.47 ± 67.98	186.19 ± 73.13	-24.28	<0.001	-4.250
>140 mg/dL at Baseline	151	226.35 ± 61.27	194.62 ± 74.90	-31.73	<0.001	-4.874

Change in the Lipid Profile

A notable improvement was observed in the lipid parameters of the patients prescribed saroglitazar. The LDL and TG levels (n = 553) demonstrated significant reductions from baseline to follow-up visit. LDL levels decreased by -6.95 mg/dL (p<0.001), and TG levels showed a significant reduction of -55.41 mg/dL (p<0.001). Similarly, the TC levels (n=54) also showed a significant lowering of -20.16 mg/dL (p=0.0049). However, changes in HDL levels (n=476) and non-HDL cholesterol levels (n=40) were not statistically significant (Table 5).

**Table 5:** Change in the lipid parameters p-value calculated using Signed Rank-Sum at 5% level of significance. **p-value at 0.05 levels of significance ***p-value significant at 0.001 level of significance. The data have been represented as N, Mean±SD. LDL: Low-Density Lipoproteins, TG: Triglycerides, HDL: High-Density Lipoproteins, TC: Total Cholesterol, non-HDL: non-High-Density Lipoproteins, SD: Standard Deviation, N: Number of patients

Parameter	N	Baseline (Mean ± SD)	Follow-up (Mean ± SD)	Mean change	p-value	z-value
LDL (mg/dL)	553	91.96 ± 39.06	85.01 ± 33.25	-6.95	<0.001^***^	-4.202
TG (mg/dL)	553	219.88 ± 101.39	164.47 ± 84.88	-55.41	<0.001^***^	-13.104
HDL (mg/dL)	476	42.46 ± 9.56	41.93 ± 9.73	-1.26	0.1019	-1.636
TC (mg/dL)	54	177.51 ± 41.78	157.35 ± 43.45	-20.16	0.0049^**^	-2.811
non-HDL (mg/dL)	40	131.08 ± 39.68	122.35 ± 44.46	-8.73	0.1447	1.459

Change in the Liver Enzyme Levels

Table 6 presents the mean changes in the AST and ALT levels among the overall study population (n=553) and in patients with diseased liver conditions (n=143). The AST and ALT showed statistically significant reductions in the overall population, with mean changes of -2.62 IU/L and -7.75 IU/L, respectively (p < 0.001). Among the patients with MASLD, the mean reduction in AST was −5.95 IU/L, while ALT decreased by −14.47 IU/L, statistically significant (p < 0.001). These changes indicate improvements in liver function. The sample size for the patients with fatty liver was smaller. However, a stability or improvement in milder cases of fatty liver was observed at follow-up.

**Table 6:** Changes in the AST and ALT levels p-value calculated using the Signed Rank-Sum test at the 5% level of significance. ***p-value significant at 0.001 level of significance. The data have been represented as N, Mean±SD AST: Aspartate Transaminase, ALT: Alanine Aminotransferase, SD: Standard Deviation, n: Number of patients

Changes in the AST and ALT levels in the overall population (n = 553)					
Parameter	Timepoint		Mean change	p-value^***^	z value
	Baseline (Mean ± SD)	Follow-up (Mean ± SD)			
AST (IU/L)	29.40 ± 11.42	26.78 ± 10.09	-2.62	<0.001	5.435
ALT (IU/L)	36.19 ± 17.95	28.24 ± 14.96	-7.95	<0.001	10.630
Changes in the AST and ALT levels in patients with diseased liver conditions (n = 143)					
AST (IU/L)	35.17 ±11.62	29.22 ± 11.03	-5.95	<0.001	6.185
ALT (IU/L)	45.70 ± 18.62	31.23±15.53	-14.47	<0.001	7.683

Safety and tolerability of saroglitazar

Renal Function Parameters

No statistically significant change was observed in the eGFR from baseline (93.97 ± 22.23 mL/min/1.73 m²) to the follow-up visit (92.15 ± 22.51 mL/min/1.73 m²) (p = 0.2734) in the overall population with available eGFR data (N=424). No significant change was observed in serum creatinine values at follow-up from baseline (N=455, p=0.1040) (Table 7).

**Table 7:** Changes in the eGFR and serum creatinine levels from baseline to follow-up visit (1) p-value calculated using a paired t-test at a 5% level of significance. (2) p-value calculated using the Signed Rank test at a 5% level of significance. (1) p-value calculated using Paired t-test at 5% level of significance. (2) p-value calculated using Signed Rank test at 5% level of significance. The data have been represented as N, Mean ± SD eGFR: Estimated Glomerular Filtration Rate, SD: Standard Deviation

Parameters	N	Baseline (Mean ± SD)	Follow-up (Mean ± SD)	Mean change	p-value	Test value
eGFR (mL/min/1.73 m²)	424	93.97 ± 22.23	92.15 ± 22.51	-1.82	0.2734^(2)^	z-value=1.095
Serum Creatinine (mg/dL)	455	0.93 ± 0.12	0.99 ± 0.64	0.06	0.1040^(1)^	t-value=-1.6290

Adverse Events

At the follow-up visit, a small proportion of patients experienced adverse events. The most frequently reported events were pyrexia (N=5, 0.90%), gastritis (N=4, 0.72%), and asthenia (N=4, 0.72%). Other reported events included vomiting, rash, and myalgia. No significant change was observed in the BMI (n=370) from baseline to follow-up visit (Table 8).

**Table 8:** Adverse events at the follow-up visit *The frequencies denote the adverse events based on the adverse events mentioned in the complaints section in the EMR. The denominator for percentages is 553. The data have been represented as N (%)

Event	N (%)*
Pyrexia	5 (0.90)
Gastritis	4 (0.72)
Asthenia	4 (0.72)
Vomiting	3 (0.54)
Rash	3 (0.54)
Myalgia	1 (0.18)

## Discussion

Metabolic diseases are a globally widespread group of interrelated conditions that often require polypharmacological approaches to manage conditions like DM, hypertension, and dyslipidemia. However, complex regimens increase drug-drug interaction risks, compromise adherence, and hinder optimal outcomes [19]. A multitarget pharmacologic approach could be advantageous for mitigating these challenges by improving efficacy and reducing the need for multiple medications [20]. This study underscores the effectiveness of saroglitazar as a comprehensive therapeutic option, particularly in the Indian population, where the burden of metabolic diseases is on the rise [5].

Saroglitazar, a dual PPARα/γ agonist, significantly reduces lipid and glycemic indicators through its strong PPARα and moderate PPARγ agonism, respectively [21]. In this study, HbA1c levels decreased by -0.71% from baseline to the follow-up visit. Patients with baseline HbA1c ≥6.5% showed a significant reduction of −0.87% (p<0.001), while those with HbA1c ≥7% and ≥7.5% had reductions of −1.05% and −1.21%, respectively. A marked decline in the FBG (-21.24 mg/dl) and PPBG (-24.28 mg/dl) was also seen, accentuating its moderate PPAR-γ agonist activity. These improvements suggest a potential role of saroglitazar in reducing endothelial aggression, a precursor to coronary disease [21, 22]. These results align with findings from the euglycemic clamp study of Jain et al., which demonstrated saroglitazar’s ability to enhance insulin sensitivity, further reinforcing its efficacy as a glucose-dependent anti-hyperglycemic agent [23]. 

Dyslipidemia patterns in Indian adults typically feature low HDL, high TGs, high cholesterol, and high LDL [24]. The prescription of saroglitazar resulted in a statistically significant reduction in the lipid parameters, including TG (−55.41 mg/dL), LDL (−6.95 mg/dL), and TC (−20.16 mg/dL). These effects are attributed to PPARα agonism, which enhances hepatic fatty acid oxidation and lipid profile improvement [15]. Our findings align with a previous observational study on saroglitazar in patients with T2DM and MASLD, where a significant TG reduction was noted after 12 weeks of its initiation [25]. Several clinical trials, including the GLIDDER and PRESS V studies, demonstrated similar improvements in lipid profiles [26,27]. Importantly, saroglitazar also showed efficacy in patients unresponsive to statin therapy, as reported in PRESS VI, positioning it as a valuable option for hypertriglyceridemia in patients with type 2 diabetes mellitus (T2DM) [13]. These findings highlight the dual role of saroglitazar in reducing hypertriglyceridemia and enhancing insulin sensitivity along with β-cell function by reduction in gluco-lipotoxicity via PPARγ agonism, garnering interest in its use for MASLD and related metabolic conditions [28].

Elevated transaminases indicate ongoing liver cell damage and are frequently abnormal in patients with MASLD. Although transaminase levels don't correlate with liver fibrosis, they are regularly monitored in MASLD patients in both routine practice and clinical drug trials due to their widespread availability and low cost [29]. In the current study, significant reductions in AST and ALT levels were observed in the overall population as well as in the patients with diseased liver conditions, suggesting that saroglitazar is efficacious in attenuating liver conditions [30]. These findings are consistent with the previous studies, such as the SVIN trial, which reported a pronounced ALT reduction with saroglitazar, and that of Goyal et al., where a significant decrease in ALT and AST after 24 weeks of therapy was documented [31,28]. The observations are also concurrent with those of the EVIDENCES IV and GLIDDER studies and also with the study conducted by Advanced Medical Research Institutes (AMRI), Kolkata, India, which reported a prominent decrease in the liver enzymes [29,26,17]. Further, findings of Chaudri et al. [17] reported pronounced changes in the controlled attenuation parameter (CAP) and liver stiffness measurement (LSM) scores on FibroScan. However, in our study, the sample size for evaluating the CAP and LSM scores was very small.

Adverse events like pyrexia, gastritis, asthenia, etc., were reported in our study, comparable to a clinical study on saroglitazar for a course of 12 weeks in which incidences of pyrexia, dyspepsia, and gastritis with mild and moderate intensity were reported [13]. A prospective, observational study reported dyspepsia and fatigue with saroglitazar [28]. In this line, it is intriguing that the well-known side effects of PPAR agonists were not observed in the present study. Additionally, there was no significant change in the BMI across visits, further supporting the overall tolerability of the drug. Moreover, saroglitazar showed a non-significant change in serum creatinine and eGFR levels, suggesting minimal impact on renal function, which might be due to its non-renal route of excretion [32]. It was also evidenced from the results of Vuppalanchi et al. that the pharmacokinetic profile of Saroglitazar Magnesium 2 mg was comparable in patients with severe renal impairment and those with normal renal function. This elucidates that renal impairment has minimal impact on the pharmacokinetics of saroglitazar, indicating the non-requirement of dose adjustment [33].

A considerable number of patients were seen to be taking antidiabetic, antihyperlipidemic, and antihyperglycemic medications along with saroglitazar. This is in concurrence with the observations of Goyal et al., where a similar pattern of concomitant medications was recorded [28].

The study has certain limitations. The number of patients with the data available on fatty liver grading was low, limiting the generalizability and statistical strength of the findings. The lack of a control group also limits the causal inference, and the missing data was not imputed, which may affect the completeness of the findings. Moreover, different drugs for various underlying comorbid conditions were prescribed to the patients, which could act as a confounding factor for the study. These concomitant medications may have influenced clinical parameters, independent of saroglitazar.

The results of this study underscore the potential of saroglitazar to address the intricate challenges of metabolic diseases, reducing the need for polypharmacy. This is the first EMR-based retrospective, real-world study evaluating the effectiveness, safety, and tolerability of saroglitazar in Indian patients with metabolic conditions. Further studies with a longer duration and larger sample size are warranted to establish long-term effectiveness and tolerability.

## Conclusions

In conclusion, this real-world EMR-based study highlights the multifaceted therapeutic benefits of saroglitazar in metabolic disorders, including T2DM, dyslipidemia, and MASLD. Saroglitazar leads to significant improvement in the glycemic, lipid, and liver enzyme parameters, underscoring its dual PPARα/γ agonist action. Its favorable safety and tolerability profiles and potential to reduce polypharmacy burden make it a promising option for comprehensive metabolic management. Therefore, saroglitazar could be a potentially good therapeutic option for mitigating metabolic diseases in Indian patients.
